# Office-Based Deep Sedation for Pediatric Ophthalmologic Procedures Using a Sedation Service Model

**DOI:** 10.1155/2012/598593

**Published:** 2012-03-14

**Authors:** Kirk Lalwani, Matthew Tomlinson, Jeffrey Koh, David Wheeler

**Affiliations:** ^1^Department of Anesthesiology and Peri-operative Medicine and Department of Ophthalmology, Oregon Health & Science University, 3181 SW Sam Jackson Park Road, Portland, OR 97239, USA; ^2^Doernbecher Children's Hospital and Casey Eye Institute, Oregon Health & Science University, 3181 SW Sam Jackson Park Road, Portland, OR 97239, USA; ^3^Legacy Health, University of Utah, Salt Lake City, UT 84112, USA

## Abstract

*Aims*. (1) To assess the efficacy and safety of pediatric office-based sedation for ophthalmologic procedures using a pediatric sedation service model. (2) To assess the reduction in hospital charges of this model of care delivery compared to the operating room (OR) setting for similar procedures. *Background*. Sedation is used to facilitate pediatric procedures and to immobilize patients for imaging and examination. We believe that the pediatric sedation service model can be used to facilitate office-based deep sedation for brief ophthalmologic procedures and examinations. *Methods*. After IRB approval, all children who underwent office-based ophthalmologic procedures at our institution between January 1, 2000 and July 31, 2008 were identified using the sedation service database and the electronic health record. A comparison of hospital charges between similar procedures in the operating room was performed. *Results*. A total of 855 procedures were reviewed. Procedure completion rate was 100% (C.I. 99.62–100). There were no serious complications or unanticipated admissions. Our analysis showed a significant reduction in hospital charges (average of $1287 per patient) as a result of absent OR and recovery unit charges. *Conclusions*. Pediatric ophthalmologic minor procedures can be performed using a sedation service model with significant reductions in hospital charges.

## 1. Introduction

 Pediatric patients frequently undergo brief ophthalmologic examinations or procedures for the management of such conditions as congenital glaucoma, cataracts, and obstructed nasolacrimal ducts. Frequently, these children are unable to cooperate as a result of their young age or the uncomfortable nature of the procedure [1]. These cases have traditionally been carried out in the operating room (OR) under general anesthesia. Anesthesia in the OR setting incurs extra hospital charges and some degree of inconvenience (e.g., scheduling, recovery time) for the surgeon, patient, and the patient's family. Thus, an alternative model for providing sedation or anesthesia in a location outside of the OR (a sedation service model) may be advantageous [2]. The sedation service model has the following benefits: (1) procedure seems less “invasive” to the parents and patients, (2) process is more convenient for surgeons as it can be performed as part of an outpatient clinic session, (3) the office location avoids unnecessary utilization of valuable operating room resources and block time, and (4) may result in fiscal benefits to the healthcare system. 

 We believe that the pediatric sedation service model can deliver these benefits for brief ophthalmologic procedures and examinations. Although there have been reports of using sedation for ophthalmologic examinations under anesthesia (EUAs), there is little information about doing a wider range of procedures outside of the OR. In a large study on the safety of pediatric sedation, only 44 of the 20,950 non-radiological procedures were ophthalmological in nature [3]. It has been our institutional model to perform these brief, minimally invasive ophthalmologic procedures with the help of the pediatric sedation service. The vast majority of these procedures are performed in a clinic treatment room under propofol deep sedation. Other studies have shown the sedation service model to be advantageous so long as there is adequate preparation and a sedation protocol in place [4, 5].

We present data from our experience with pediatric ophthalmologic procedures in the office setting as an alternative to performing these types of procedures in the OR.

## 2. Materials and Methods

 After receiving institutional review board approval, all patients undergoing office-based ophthalmologic procedures outside of the operating room at Oregon Health & Science University's Casey Eye Institute between January 1, 2000 and July 31, 2008 were identified using the sedation service database and the electronic medical record. The data collected from the 591 patients' medical records included demographics, weight, ASA status, procedure/s, medications, sedation time, and complications. A comparison analysis of hospital charges for similar procedures (OR versus sedation service) was then performed based on information obtained from the institutional billing office.

 A standard sedation service protocol was followed for all procedures. This protocol includes medical prescreening (by a sedation nurse for routine cases and by an anesthesiologist for patients with complex medical histories or difficult airways), history and physical, and an appropriate fasting interval. Patients deemed to be at high risk for deep sedation in a remote office-based setting were either sedated or anesthetized in the operating room suites by the anesthesiologists assigned to the operating rooms; these patients are not included in this series. On arrival, Ela-Max cream is applied to the child's hands or feet (30 minutes before the procedure), and IV access is obtained. Following the application of standard monitoring equipment, the patient is sedated by one of the team's pediatric anesthesiologists. Propofol was used as the primary agent for induction and maintenance of deep sedation (typically 1.0–5.0 mg/kg). Deep sedation is maintained with a propofol infusion or incremental boluses, with or without small doses of opioids like alfentanil. Alfentanil was titrated in small doses for painful procedures (nasolacrimal duct probing, speculum insertion in the eye, etc.) to minimize the total dose of propofol administered to maintain immobility. The level of sedation, airway patency, and adequacy of ventilation were continuously monitored by the anesthesiologist; continuous pulse oximetry and noninvasive blood pressure monitoring was utilized. In addition, the sedation nurses used the Ramsay sedation scale to monitor and record the level of sedation. Patients were recovered at the site of sedation in the office or clinic, by a trained sedation nurse experienced in pediatric sedation and pediatric intensive care with continuous pulse oximetry and noninvasive BP monitoring. During recovery, the patients were given IV fluids and discharged home when standard discharge criteria were met.

## 3. Results

### 3.1. Demographics and Procedure Data

 A total of 855 procedures (591 patients, 264 repeat procedures) were identified, of which 43% were female and 57% male. Mean (SD) age was 10.4 (4.6), mean weight was 17.9 (12.4) kg, and mean total propofol dose was 4.8 mg/kg (SD 5.2), (95% C.I. 4.4, 5.1). The mean dose of alfentanil was 13.6 mcg/kg (SD 9.8). Mean procedure time was 31.5 (20.6) minutes, and the mean recovery time was 11.8 (13.4) minutes. 87% of patients were classified as ASA physical status 1 or 2 (Tables 1, 2 and 3).

### 3.2. Procedures

 The most common procedures performed were electroretinography (ERG) (38.6%), examination under anesthesia or measurement of intraocular pressure (38.3%), nasolacrimal duct probing/silicone tube insertion (9.4%), suture removal (2.6%), and miscellaneous (11.1%) (Table 4). 

### 3.3. Efficacy

The procedure completion rate was 100% (C.I. 99.62–100). There were no aborted procedures or unplanned admissions. Two patients were cancelled prior to sedation (respiratory infection, failed IV access), and 11 (0.01%) patients had a prolonged recovery (>1 hour) (Table 5).

### 3.4. Safety

 There were no serious complications, and 13.9% had minor complications such as transient oxygen desaturation (8.5%), airway obstruction (3.6%), and apnea (0.6%), all of which are common with propofol deep sedation in children (Table 6). Based on recorded data, no clinically significant hypotension (requiring any intervention such as a fluid bolus, or >20% deviation from baseline) was recorded in this series of procedures.

One patient fell off the table following a seizure during ERG in the dark but was unharmed. One patient experienced bradycardia associated with an irregular pulse that resolved with atropine treatment following corneal electrode placement. Four patients lost their IV as a result of a sharp withdrawal reflex secondary to the discomfort of propofol administration; these were all successfully replaced in the dark prior to an ERG. Other events included equipment difficulties related to the infusion pump (*n* = 2), and a photostimulator malfunction (*n* = 1) during the procedure; in the latter case, the procedure was completed with the adult photostimulator.

### 3.5. Interventions

 The most common interventions for the above-mentioned minor complications were supplemental oxygen (12.6%), jaw thrust (4.8%), and bag-mask ventilation (1%). All patients recovered completely following these interventions.

### 3.6. Hospital Charges

Our practice has evolved such that the procedures described here are rarely performed in the OR. This makes it nearly impossible to directly compare hospital charges for procedures performed in and out of the OR. Therefore, a hypothetical model was developed based on a 30-minute case length for the procedure and the least possible cost for other perioperative charges (Table 7). Professional fees for similar cases would be identical. Differences in medication costs are insignificant compared to hospital charges. Based on this model, procedures performed outside of the OR resulted in a reduction of hospital charges of nearly $1300 per case by reducing facility-related charges by more than 70%. Results of other studies [6, 7] in which similar sedation techniques were used showed significant reduction in charges ($1250–$4849 per patient) by avoiding operating room and recovery unit charges. Our institution utilizes an identical model to these studies.

## 4. Discussion

 The results of this series of patients demonstrate the feasibility and efficacy of providing office-based sedation for ophthalmological procedures for children in a hospital setting with a motivated and skilled pediatric sedation service. Pediatric patients undergoing a variety of brief ophthalmologic procedures can be safely anesthetized outside of the OR [3, 8].

### 4.1. Efficacy and Safety

All procedures were completed successfully with no major complications. Side effects related to deep sedation with propofol (transient oxygen desaturation, airway obstruction) that respond to simple interventions such as blow by oxygen, head repositioning, and so forth were similar to the numerous published studies of propofol deep sedation for pediatric procedural sedation. Adverse events and complications rarely occurred, despite many procedures traditionally deemed “unsuitable” for nonintubated patients [8]. For example, during nasolacrimal duct probing, the surgeons were aware that patients were not intubated and limited the volume of saline irrigation; they also typically add a drop of sodium fluorescein in the eye for easy nasal detection; in addition, a soft suction catheter is occasionally placed in the oral cavity and left on continuous suction by the anesthesiologist.

Given the low incidence of major complications during pediatric sedation, prospective evaluation of a large sample size of this subset of patients in the tens of thousands would be necessary to establish “safety” of sedation in order to avoid a Type 2 error. Since the propofol deep sedation technique is very similar to that in the published literature, and all pediatric deep sedation has been performed by trained pediatric anesthesiologists with the assistance of skilled pediatric sedation nurses since the inception of the service at our institution in 1995, it would not be unreasonable to infer that given the quality and experience of the sedation service the rate of major complications at our institution is likely to be extremely low. The pediatric sedation research consortium has successfully published data attesting to the low incidence of complications of this practice in high quality, motivated sedation services [3].

### 4.2. Sedation Service Model

Movaghar et al. demonstrated the reduction in procedure duration and comparable efficacy in a small series of patients undergoing nasolacrimal duct probing with propofol [8]. In addition, several opportunity costs favor the sedation service model; less anesthesia time is required; parents lose less time from work; the ophthalmologist can perform these minor procedures while in clinic; the OR is freed up for more complex cases requiring the sterile environment.

### 4.3. Limitations

 The limitations of this study are its retrospective nature and the inability to generalize the findings to institutions with less-motivated or experienced sedation services or providers. This large series of patients demonstrate the feasibility and advantages of providing deep sedation for pediatric ophthalmologic procedures in an office-based setting with a pediatric sedation service model. Avoidance of the side effects of general anesthesia such as sore throat and postoperative nausea and vomiting contribute to a speedy recovery and discharge home. This has been well accepted at our institution by patients, parents, and surgeons.

## 5. Conclusions

Office-based ophthalmologic procedures in children can be performed using a pediatric sedation service model to deliver deep sedation. Office-based sedation is convenient, provides significant advantages for patients, families, and surgeons, and frees up operating room resources. Significant reductions in hospital charges can also be achieved.

## Figures and Tables

**Table 1:** Demographic and other patient data.

Demographics		
Gender	Number	%
Male	337	57
Female	254	43

	Mean	SD

Age (years)	10.4	4.6
Weight (kg)	17.9	12.4

**Table 2:** ASA physical status.

ASA status	Number	%
(1)	294	34
(2)	455	53
(3)	105	12
(4)	1	0.117
(5)	0	0
(6)	0	0

**Table 3:** Procedure details.

Procedure data		
	Total	Repeat cases
Procedures	855	264

	Mean	SD

Procedure time (min)	31.5	20.6
Total propofol dose (mg/kg)	4.8	5.2
Recovery time (min)	11.8	13.4

**Table 4:** Most common procedures performed.

Procedure	*n*	%
ERG	330	38.6
EUA/IOPM	327	38.3
NLD probe/ tube insertion	81	9.4
Suture removal	22	2.6
Other	95	11.1

**Table 5:** Procedure efficacy and safety.

Procedure efficacy	*n*	%
Procedures completed	855	100
Delayed discharges	11	0.01
Unplanned admissions	0	0.0
Aborted procedures	0	0.0

**Table 6:** Complications observed (all minor).

Complication	Number	%
Major complications	0	0
Transient oxygen desaturation	51	8.6
Airway obstruction	21	3.6
Apnea	4	0.6

**Table 7:** Average reduction in hospital charges compared to the OR setting.

Charges	In OR	Out of OR
Pre-op	$56	$0
OR/procedure Room	$1,379	$230
Recovery	$350	$268

Total	$1,785	$498
